# A Novel Computational Model for Predicting microRNA–Disease Associations Based on Heterogeneous Graph Convolutional Networks

**DOI:** 10.3390/cells8090977

**Published:** 2019-08-26

**Authors:** Chunyan Li, Hongju Liu, Qian Hu, Jinlong Que, Junfeng Yao

**Affiliations:** 1 School of Informatics, Xiamen University, Xiamen 361005, China; 2 Graduate School, Yunnan Minzu University, Kunming 650504, China; 3 College of Information Technology and Computer Science, University of the Cordilleras, Baguio 2600, Philippines

**Keywords:** disease, microRNA, heterogeneous, graph, convolution network, negative sampling, cross validation

## Abstract

Identifying the interactions between disease and microRNA (miRNA) can accelerate drugs development, individualized diagnosis, and treatment for various human diseases. However, experimental methods are time-consuming and costly. So computational approaches to predict latent miRNA–disease interactions are eliciting increased attention. But most previous studies have mainly focused on designing complicated similarity-based methods to predict latent interactions between miRNAs and diseases. In this study, we propose a novel computational model, termed heterogeneous graph convolutional network for miRNA–disease associations (HGCNMDA), which is based on known human protein–protein interaction (PPI) and integrates four biological networks: miRNA–disease, miRNA–gene, disease–gene, and PPI network. HGCNMDA achieved reliable performance using leave-one-out cross-validation (LOOCV). HGCNMDA is then compared to three state-of-the-art algorithms based on five-fold cross-validation. HGCNMDA achieves an AUC of 0.9626 and an average precision of 0.9660, respectively, which is ahead of other competitive algorithms. We further analyze the top-10 unknown interactions between miRNA and disease. In summary, HGCNMDA is a useful computational model for predicting miRNA–disease interactions.

## 1. Introduction

MicroRNAs (miRNAs) are small, non-coding RNAs that play an important role in inhibiting the expression of target mRNAs at the post-transcriptional level with vital molecular functions and participate in almost all important life processes. Specifically, miRNAs regulate target genes and result in cleavage or translation inhibition in target mRNAs [1,2,3]. Currently, over 30,000 miRNAs within approximately 200 species have been identified [4]. An increasing number of empirical evidence shows that it is important for disease development and progression and that miRNAs may be positive regulators at post-transcriptional level [5,6]. Herein, miRNAs clearly have a critical impact on human diseases. Predicting the interactions between diseases and miRNAs is a vital problem [7]. Computationally predicting diseases and miRNAs accelerates the identification of real disease-associated miRNAs. Recent human protein–protein interactions (PPI) network modeling suggests that network-based approaches offer possibilities to identify miRNA–disease interactions.

Over the past few years, numerous computational approaches have been built for predicting miRNA–disease associations [8,9,10,11,12,13,14,15,16], mainly in two categories: Similarity-based measure approaches and machine learning approaches. Nevertheless, several methods that use machine learning are essentially based on similarity measures and matrix factorization. Bipartite network for predicting miRNA–disease association, abbreviated as BNPMDA, is a novel model of prediction which integrates miRNA and disease similarities using the known miRNA–disease interactions. However, BNPMDA cannot predict diseases without any known related miRNAs [8]. Another novel method of inductive matrix completion (IMCMDA) integrates miRNA and disease similarities [9]. IMCMDA is used to forecast the missing miRNA–disease interactions based on the known miRNA–disease interactions. Global linear neighborhoods method (GLNMDA) reconstructs miRNA–disease similarity matrix, then implements label propagation to infer the latent interactions between miRNAs and diseases [10]. Yu et al. [11] proposed a method for miRNA-disease association prediction based on Matrix completion and Label Propagation (MCLPMDA), which reconstructs the miRNA–disease similarity matrix using label propagation and matrix completion. Another structural perturbation method (SPM), which is also similarity-based link prediction method is applied to predict disease-related miRNAs [12]. Meanwhile, deep learning methods have attracted considerable attention because of their high accuracy. Decagon, a method for modeling polypharmacy side effects, introduced a new graph auto-encoder that is an end-to-end deep trainable model to predict associations on multimodal graph based on graph convolutional network (GCN) [17]. In this method, different edge types are modeled separately. Decagon obtains good prediction performance. However, Decagon requires individual training for each side type. Another deep learning model, dgMDL predicts disease–gene associations by deep belief net (DBN). At first, dgMDL learns feature representations based on similarity networks by two DBNs separately; then, as a mutimodal input, a joint DBN is applied for the final representations. Finally, associations between disease and gene are predicted using the joint features [18]. However, dgMDL is based on similarity estimation and employs a two-stage pipeline, which typically consists of a separate feature extraction module and link prediction module.

The existing computational approaches to predict disease-related miRNAs have made great progress, but there are still some shortcomings. Therefore, new computational methods should be better studied to excavate the potential relationships between miRNA and disease. Compared to convolutional neural networks (CNN) and recurrent neural networks (RNN), biological information networks such as diseases and genes are more suitable for graph-based modeling. On the graph data, the graph neural network(GNN) shows superior modeling performance and computing performance [19]. Especially, graph convolutional network (GCN) [20] achieves better performance in homogeneous networks, such as classification, but GCN has not been used in heterogeneous networks to predict miRNA–disease interactions. In this paper, taking no account of similarity, we propose the heterogeneous graph convolutional networks for miRNA–disease associations prediction (HGCNMDA) model based on the known PPI to integrate four biological networks: miRNA–disease, miRNA–gene, disease–gene, and the human PPI. HGCNMDA is an end-to-end trainable model for association prediction on heterogeneous environments that can be simultaneously trained for feature extraction and link prediction. The global gene graph network is initially built based on PPI, and then the disease–gene and miRNA–gene graph network are separately built. Furthermore, cross features are extracted from different networks with the node2vec algorithm [21]. Finally, miRNA–disease interactions are predicted. HGCNDMA can thus accurately predict the interactions between disease and miRNA with the learned cross features representations in heterogeneous environments.

Our contributions to this article are as follows: 1) We propose a novel end-to-end deep learning architecture for predicting miRNA–disease associations based on heterogeneous graph networks. Graphs are directly accepted as input without the need for any preprocessing. 2) We first develop a novel heterogeneous graph convolutional HGCNMDA to extract the multi-scale characteristics of vertexes between different networks. 3) Experimental results show that our HGCNMDA is highly competitive with state-of-the-art algorithms, and significantly outperforms many other similarity measure approaches and machine learning methods for predicting miRNA–disease associations.

## 2. Materials and Methods

### 2.1. Reconstruction of Heterogeneous Networks

#### 2.1.1. The Human Protein–Protein Interactions

The human PPI network is obtained from Zitnik et al. [17]. It is produced by Menche et al. [22] and Chatr-Aryamontri et al. [23], additional PPI information from Szklarczyk et al. [24] and Rolland et al. [25] is integrated. The proteins in PPI network are mapped to corresponding genes to constitute a gene–gene association network. There are 19,081 genes and 715,612 physical interactions in total, and the edge is unweighted and undirected.

#### 2.1.2. Disease–Gene Network

Disease–gene interaction data can be gained from four public databases: The Online Mendelian Inheritance in Man (OMIM) [26], HuGE Navigator [27], PharmGKB [28], and Comparative Toxicogenomics Database (CTD) [29]. We annotate these disease terms and genes using MeSH [30] and the Entrez IDs, respectively. In total, the disease–gene network contains 51,544 disease–gene associations with 394 unique disease terms and 2673 different genes.

#### 2.1.3. miRNA–Gene Network

The known miRNA targets are downloaded to build miRNA–gene networks from miRTarBase database [31]. All of protein-coding genes are annotated according to gene Entrez ID and the symbols in the National Center for Biotechnology Information (NCBI) database [32]. In total, the miRNA–gene network contains 163,090 miRNA–gene associations with 569 miRNAs and 14,259 different genes after excluding duplicate associations.

#### 2.1.4. miRNA–Disease Network

The known human interactions data between miRNA and disease can be gained from miR2Disease [33] and HMDD v3.0 [34] databases. All disease terms were annotated by Medical Subject Headings (MeSH). After removing duplicate associations, finally, the miRNA–disease network contains 7669 associations with 394 diseases in total. 

**Figure 1:**
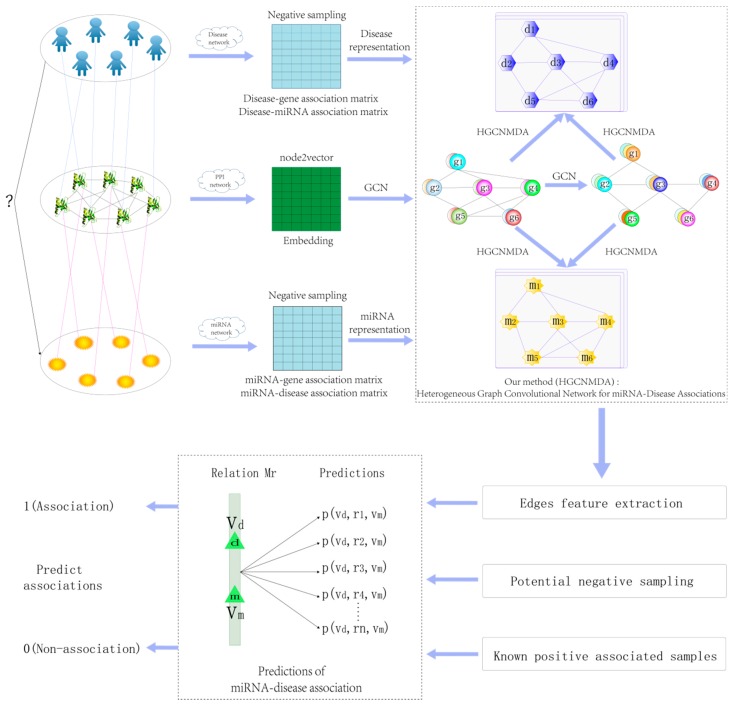
The overall structure of heterogeneous graph convolutional network for miRNA–disease associations HGCNMDA. Input graphs of arbitrary structure are first passed through graphs convolution layers where node information is propagated between neighbors. First, features in gene–gene association network are obtained using graph convolution. Secondly, disease features and miRNAs features are extracted respectively based on PPI features using HGCNMDA approach that we proposed. Lastly, the edge features between diseases and miRNAs are extracted and passed to decoder layer to train a predictive model after obtaining potential negative sampling together with known positive associated samples. Features are visualized as colors.

An adjacency matrix $A\in R^{dxm}$ about miRNA–disease associations is constructed, where d and m are denoted as the count of known diseases and known miRNAs. In the study, because HGCNMDA is based on PPI network, some disease nodes and miRNA nodes in disease–miRNA network will be removed if the genes associated with them are not in PPI network. If a disease $d_{i}$ has been confirmed to be related to an miRNA $m_{j}$, then $A_{ij}$ is equal to 1; if not, $A_{ij}$ equals 0. Figure 1 demonstrates a flowchart of HGCNMDA for heterogeneous environments.

### 2.2. Raw Feature Extraction

Besides graph structure features, latent features should be also studied for link prediction [35]. In order to learn a low dimensional latent representation/embedding for each node, latent feature methods [21,36] factorize some matrix representations of the network. Examples include the matrix factorization method [37] and stochastic block model method [38]. Recently, some network embedding techniques, such as node2vec [21] and DeepWalk [36], are also latent feature methods since they implicitly factorize some matrices [39]. It is shown that combining graph structure features with latent features can improve the performance [35,40]. In this paper, we choose the node2vec algorithm [21] to achieve latent features of the PPI network. Node2vec takes the graph and its edges and encodes the graph information in node embeddings. It performs random walk for 10 iterations and captures both local topological information and global structural information for feature extraction. Empirically feature size is set 64, 128, and 256, respectively.

### 2.3. Graph Convolution Network

Graph convolutional network (GCN) [20] obtains good performance for node classification tasks by combining local graph structure and node features. Given a graph G = (V,ℰ) with N nodes $\nu_{i}\in V$ and edges $\left(\nu_{i}\;,\;\nu_{j}\right)\;\in ℰ$, $A\in {\mathbb{R}}^{N\times N}$ is an adjacency matrix and $D_{ii}=\sum_{j}A_{ij}$ is a degree matrix. Graph convolution layer takes the following form:

(1) $$H^{\left(\ell +1\right)}=\;f\left({\tilde{D}}^{-\frac{1}{2}}\tilde{A}{\tilde{D}}^{-\frac{1}{2}}H^{\left(\ell \right)}W^{\left(\ell \right)}\right)$$

Where $\tilde{A}=A+I$ denotes the adjacency matrix of a graph plus identity matrix I, ${\tilde{D}}_{ii}$ = $\sum_{j}{\tilde{A}}_{ij}$, $W^{\left(\ell \right)}$ is a weight matrix which changes with the different value of ℓ. f(·) is an activation function. $H^{\left(\ell \right)}$ is an activation matrix in the $\ell^{\mathrm{th}}$ layer; $H^{\left(0\right)}$ is initial value. In this paper, spectral graph convolution is used [20]. It is defined as the multiplication of a signal $x\in {\mathbb{R}}^{N}$ with a filter $g_{\theta }$ = *diag*(θ) parameterized by $\theta \in {\mathbb{R}}^{N}$ in the Fourier domain, showed as following:

(2) $$g_{\theta }*x=Ug_{\theta }U^{T}x$$

(3) $$L=I-D^{-\frac{1}{2}}AD^{-\frac{1}{2}}\;=U\Lambda U^{T}$$

Here, U is the matrix of eigenvectors of L which is normalized, Λ is its eigenvalues, and $U^{T}x$ is the Fourier transform of x. Because Equation (3) is computationally expensive, computing the eigen decomposition of Laplacian L might be time-consuming and expensive for big graphs [20]. We can get its approximate value using a truncated expansion according to Chebyshev polynomials $T_{k}\left(x\right)$ up to $K^{\mathrm{th}}$ order [41]:

(4) $$g_{\theta^{\prime }}\left(\Lambda \right)\;\approx \overset{K}{\underset{k=0}{\sum }}{\theta^{\prime }}_{k}T_{k}\left(\tilde{\Lambda }\right)$$

(5) $$g_{\theta^{\prime }}*x\;\approx \overset{K}{\underset{k=0}{\sum }}{\theta^{\prime }}_{k}T_{k}\left(\tilde{L}\right)x$$

(6) $$\tilde{L}=\;\frac{2}{\lambda_{\max}}L-I$$

(7) $$T_{k}\left(x\right)=\;2xT_{k-1}\left(x\right)-{\;T}_{k-2}\left(x\right)$$

Where ${\theta^{\prime }}_{k}$ is a Chebyshev coefficient vector. $\lambda_{\max}$ is the maximum eigenvalue of graph Laplacian *L*. $T_{0}\left(x\right)=1{\;\mathrm{and}\;T}_{1}\left(x\right)=x$ are the initial values of Chebyshev polynomials. So, Equation (5) can be written as follows:

(8) $$g_{\theta^{\prime }}*x\;\approx {\theta^{\prime }}_{0}\;x+{\theta^{\prime }}_{1}\left(L-I\right)x=\;{\theta^{\prime }}_{0}\;x-{\theta^{\prime }}_{1}D^{-\frac{1}{2}}AD^{-\frac{1}{2}}\;x$$

(9) $$g_{\theta^{\prime }}*x\;\approx \theta \left(I+D^{-\frac{1}{2}}AD^{-\frac{1}{2}}\right)x$$

Where $\theta ={\theta^{\prime }}_{0}=-{\theta^{\prime }}_{1}$. In the next section, we will introduce heterogeneous graph convolutional HGCNMDA model, which is based on the GCN model.

### 2.4. Heterogeneous Graph Convolutional HGCNMDA Approach

Predicting disease–miRNA associations is modeled as a multirelational link prediction problem among some different graphs according to the encoding protein, disease, miRNA, and relationships among them. The proposed HGCNMDA method is based on the PPI network to predict disease-related miRNAs. Notice that the miRNA–disease interactions are limited to only those that are linked to genes of the PPI network. Given a graph G = (V,ℰ) with N nodes $\nu_{i}\in V$, the relation edge $\left(\nu_{i},\;r,\;\nu_{j}\right)$ indicates an association between node $\nu_{i}$ and node $\nu_{j}$. The task is to predict possible edges between disease and miRNA. Therefore, we propose a non-linear, heterogeneous multilayer graphs convolutional networks model HGCNMDA which is an end-to-end model based on graph G.

#### 2.4.1. HGCNMDA Convolution Layer and Negative Sampling

We will first describe the HGCNMDA convolution layer. In the PPI network, convolutional operations for each layer are performed which take the PPI graph data and the additional node2vec feature vectors as input, as expressed in Equation (10). Then, in order to gain miRNA–gene cross features and disease–gene cross features, each convolutional result of each layer in PPI network as an input multiplies an adjacency matrix (including miRNA–gene adjacency matrix and disease–gene adjacency matrix) and a trainable kernel parameter matrix $W_{mg}$ or $W_{dg}$, which is described in Equation (11) and Equation (12). In each layer, HGCNMDA propagates potential node features through edges of the graphs while taking into account different network structure. Finally, the convolution results of each layer are synthesized and the average of them is obtained as the final result for feature extraction.

(10) $$H_{gg}{}^{\left(\ell +1\right)}=\;f({\tilde{D}}_{gg}{}^{-\frac{1}{2}}{\tilde{A}}_{gg}{\tilde{D}}_{gg}{}^{-\frac{1}{2}}H_{gg}{}^{\left(\ell \right)}W_{gg}{}^{\left(\ell \right)})$$

(11) $$H_{mg}{}^{\left(\ell +1\right)}=f\left({\tilde{A}}_{mg}H_{gg}{}^{\left(\ell +1\right)}W_{mg}{}^{\left(\ell +1\right)}\right)$$

(12) $$H_{dg}{}^{\left(\ell +1\right)}=f\left({\tilde{A}}_{dg}H_{gg}{}^{\left(\ell +1\right)}W_{dg}{}^{\left(\ell +1\right)}\right)$$

**Figure 2:**
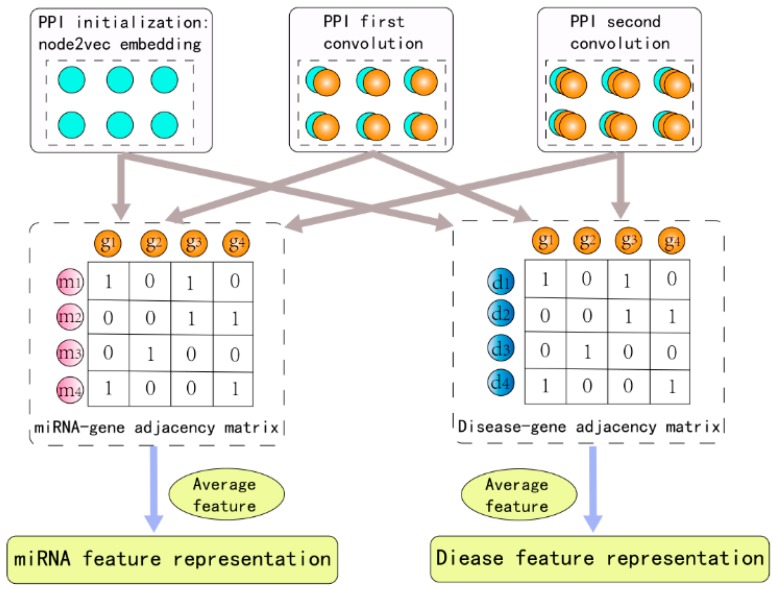
The pipeline of HGCNMDA graph convolution layer. In order to obtain miRNA feature representation and disease feature representation, the raw feature of PPI-based and convolutional result of each layer in the PPI network is as an input to miRNA–gene network and disease–gene network, respectively.

Where ${\tilde{A}}_{gg}=\;A_{gg}+\;I_{gg}$ means that the adjacency matrix of a graph plus identity matrix, ${\tilde{D}}_{ii}$ = $\sum_{j}{\tilde{A}}_{ij}$ is a degree matrix, f is an activation function, for instance, relu(·) and tanh(·). Here, $H_{gg}{}^{\left(\ell +1\right)}$ is the matrix of activations in the ${\left(\ell +1\right)}^{\mathrm{th}}$ layer in the PPI network. Analogously, $H_{mg}{}^{\left(\ell +1\right)}$ and $H_{dg}{}^{\left(\ell +1\right)}$ represent matrices of activations in the ${\left(\ell +1\right)}^{\mathrm{th}}$ layer in miRNA–gene network and disease–gene network respectively. Obviously, $H_{mg}{}^{\left(\ell +1\right)}$ and $H_{dg}{}^{\left(\ell +1\right)}$ are associated with $H_{gg}{}^{\left(\ell +1\right)}$. A deeper model can be built by adding multiple layers with proper activation functions. To obtain the final embedding $Z_{m}$(miRNA node $\nu_{m}$) and $Z_{d}$(disease node $\nu_{d}$), we compute their representation as $Z_{m}=$ $H_{mg}{}^{\left(K\right)}$ and $Z_{d}=\;H_{dg}{}^{\left(K\right)}$. The pipeline of HGCNMDA is schematically depicted in Figure 2.

Taking into account the fact that negative samples do not exist in all of the databases, our method similarly to the previous method in [18,42] and the reliable negatives is a subset which is collected from the unknown relationships as potential negative samples [43]. However, our negative sample sampling method is different from them. Here, our goal is to find more likely negative links between disease and miRNA nodes. First, in our experiment, there are 7040 known positive samples for the training of the neural network which are associated with 390 diseases and 567 microRNAs. Figure S1 shows all known positive sample distribution. Then, let $\psi_{avg}$ denote the average feature vector for all of positive samples, and $dis_{avg}$ denote the average distance.

(13) $$\psi_{avg}=\frac{1}{n}\underset{\begin{array}{c} 0\leq \;i\;\leq \;n\\ \left(j,\;k\right)\in i \end{array}}{\sum }f_{i}\left(Z_{d}^{j},Z_{m}^{k}\right)$$

(14) $$dis_{avg}=\frac{1}{n}\underset{\begin{array}{c} 0\leq \;i\;\leq \;n\\ \left(j,\;k\right)\in i \end{array}}{\sum }dis_{i}\left(Z_{d}^{j},Z_{m}^{k}\right)$$

Where n is the count of edge, $Z_{d}^{j}$ is the feature vector of disease j, $Z_{m}^{k}$ is the feature vector of miRNA k, $f_{i}\left(Z_{d}^{j},Z_{m}^{k}\right)$ is the feature vector of edge i, and (j, k) is the edge i. Similarly, $dis_{i}\left(Z_{d}^{j},Z_{m}^{k}\right)$ is the distance of edge i. For an unknown link u, $dis_{u}$ denotes the distance between u and $\psi_{avg}$. Here, two sets, “negative_dis” and “negative_edge”, need to be maintained, if the distance $dis_{u}$ > $dis_{avg}$, add $dis_{u}$ to “negative_dis” set; meanwhile, add disease node and miRNA node ($Z_{d}^{j}$, $Z_{m}^{k}$) where (j, k)∈u to “negative_edge” set. Obviously, the length of the two sets is the same. The order of all samples needs to be disrupted, including positive and negative samples, before making predictions. Because HGCNMDA employs one pipeline for extracting features and predicting links simultaneously, in the framework of tensorflow in this paper, negative sampling is handled in form of tensor. But, tensorflow can’t shuffle the tensor. In order to solve this problem, we executed a trick, firstly, shuffling the index of the tensors, and then re-indexing the tensors using the shuffled index. In contrast, in our experiment, we chose the maximum distance of the first K to be our negative links samples, because the elements in the “negative_dis” and “negative_edge” sets are one-to-one, by indexing, the true negative samples can be obtained in the “negative_edge” set. In our experiment, K is the same as the number of positive samples. So, the training dataset contains 2K samples in total.

#### 2.4.2. Edge Features Extraction

So far, we introduced HGCNMDA graph convolution layer. The layer maps each node of different network to an embedding. As a vector representation $Z_{i}\in {\mathbb{R}}^{d}$, here, d denotes the embedding dimensionality of node i. In this section, we will demonstrate the edge (between disease and miRNA) features extraction component of HGCNMDA. In particular, the method scores a $\left(\nu_{i},\;r,\;\nu_{j}\right)$-triple under the mapping of function *g*. The score $g(\nu_{i},\;r,\;\nu_{j})$ means how likely it is that disease $\nu_{i}$ and miRNA $\nu_{j}$ share association through a relation r. Here, using embeddings of nodes *d* and *m* returned by HGCNMDA graph convolution layer $Z_{d}$ and $Z_{m}$, a candidate edge $\left(\nu_{d},\;r,\;\nu_{m}\right)$ is predicted through a factorized operation [17,44,45]:

(15) $$g\left(\nu_{d},\;r,\;\nu_{m}\right)\;=\;Z_{d}M_{r}Z_{m}{}^{T}$$

Where $Z_{d}$ and $Z_{m}$ represent an embedding of disease and miRNA, respectively. $M_{r}$ is the relation-type-specific disease–miRNA parameter matrix. Then, a sigmoid function σ computes probability of edge ($\nu_{d},\;r,\;\nu_{m}$):

(16) $$p_{r}^{dm}\;=\;\sigma (g\left(\nu_{d},r,\nu_{m}\right))\;=\;\frac{1}{1+{ℯ}^{-g\left(\nu_{d},r,\nu_{m}\right)}}$$

Next, we shall describe how to optimize the HGCNMDA model and related parameters.

#### 2.4.3. HGCNMDA Model Training

We needed a loss function when training model. Here, a cross entropy loss was applied to optimize model parameters:

(17) $$loss_{ij}\;=\;-Y_{ij}*logp_{ij}-\left(1-Y_{ij}\right)log\left(1-p_{ij}\right)$$

Taking all edges into account, the final loss function in HGCNMDA was:

(18) $$ℒ=\;\underset{\left(\nu_{d},r,\nu_{m}\right)\epsilon ℛ}{\sum }loss_{ij}$$

**Figure 3:**
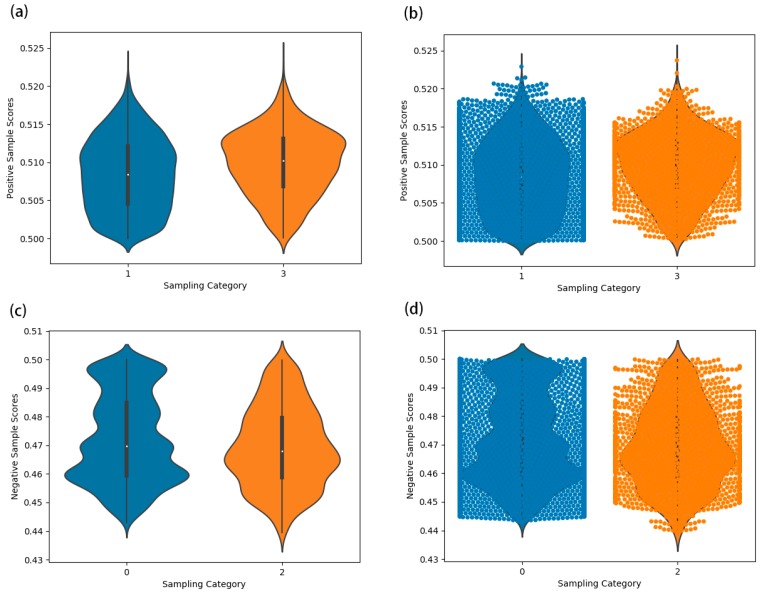
Data distributions of predicted positive and negative correlation in training set and test set in terms of violin plot and swarm plot. (**a**) Shows data distribution of predicted positive correlation for training set and testing set in terms of violin plot. (**b**) Shows a mixed graph of violin plot and swarm plot. (**c**) Shows data distribution of predicted negative correlation for training set and testing set in terms of violin plot. (**d**) Adds swarm plot on the basis of (**c**), which is similar to (**b**).

HGCNMDA classification was considered as a binary-classes problem to predict miRNA–disease associations. To avoid over-fitting, we employed the L2 regularized method to HGCNMDA’s weights by adding weight decay during training. The weight of L2 regularization term had an effect on model training, therefore, the model needed to be cross validated. Here, we implemented the 5-fold cross-validation to gain average performance. Considering training iterations, we trained 100 epochs using the Adam optimizer [46] with a learning rate of 0.001 to optimize the model. Weights were initialized using those described in [47]. Meanwhile, node feature vectors were normalized. HGCNMDA is a heterogeneous graph networks, where feature extraction and link prediction are employed by one pipeline simultaneously instead of a two-stage pipeline that consists of a feature picking-up model and an association predictive model, and the two models are trained separately. Data distributions of predicted positive and negative correlation in training set and test set were demonstrated in Figure 3. As showed in the sampling category in the violin plot, ‘0’ represents the prediction of negative correlation data distribution for training set, ‘1’ represents the prediction of positive correlation data distribution for training set, ‘2’ represents the prediction of negative correlation data distribution for testing set and ‘3’ represents the prediction of positive correlation data distribution for testing set. Here, the violin plot displayed the distribution of data across two categorical variables in order to compare those distributions. Its axis is represented by a small box chart. The point at the center of the box denotes the median. The width of the violin plot represents frequency (density of points). A swarm plot is a good complement to a violin plot, since it shows all observations along with some representation of the underlying distribution. Obviously, the points are along the categorical axis. From the distributions, positive prediction scores were mainly concentrated around between 0.503 and 0.513 in training set, between 0.508 and 0.513 in testing set. At the same time, negative prediction scores were mainly concentrated around between 0.458 and 0.462 in training set, between 0.464 and 0.469 in testing set. Therefore, we can clearly see the distribution of all the predicted data.

GCN model is a special form of Laplacian smoothing to some extent. But, over-smoothing will make the features indistinguishable and hurt the classification accuracy [48]. In this study, we specifically set two hidden layers for graph convolution with 32 hidden units for each layer.

## 3. Results

### 3.1. Overall Performance

**Figure 4:**
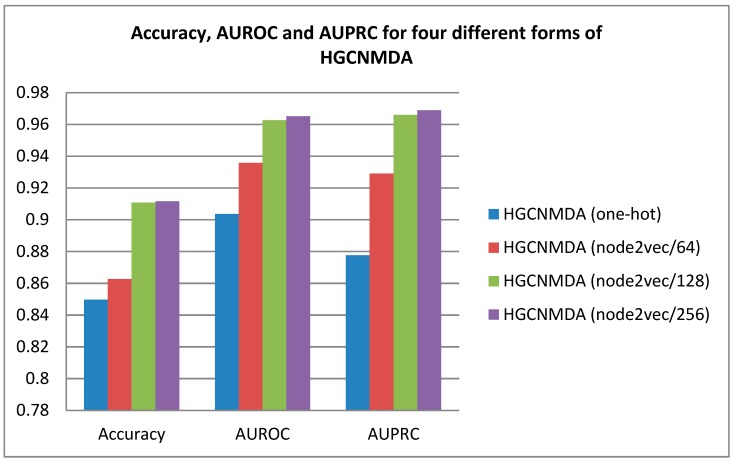
Average performance values of accuracy, area under receiver operating characteristics ROC curve (AUROC) and area under precision-recall curve (AUPRC) for disease–miRNA associations prediction for one-hot, node2vec/64, node2vec/128 and node2vec/256, respectively.

The dataset was randomly split into three subsets: Training set (80%), validation set (10%), and testing set (10%). The average area under the receiver operating characteristics (ROC) curve (AUROC) and area under precision-recall curve (AUPRC) obtained from testing sets were used to evaluate the overall performance of the model in 5-fold cross-validation in order to remove the influence of the random splitting and L2 regularization. According to 5-fold cross-validation, the total dataset was randomly divided into five mutually exclusive parts. Each part took turns to be selected as test set and the remaining four parts were applied as training set. In our experiment, we compared one hot featureless with node2vec. Meanwhile, node2vec was assigned different feature dimensions of 64, 128, and 256, marked as node2vec/64, node2vec/128, and node2vec/256, respectively. Figure 4 shows the average accuracy, AUROC scores and AUPRC scores for our method in terms of one-hot, node2vec/64, node2vec/128, and node2vec/256, respectively. Specifically, the value of AUROC is greater than 0 and less than 1, and the larger the AUROC value, the better the predictive result. As depicted in Figure 4, HGCNMDA obtained AUROC values of 0.9036, 0,9358, 0.9626, and 0.9651 in one-hot, node2vec/64, node2vec/128, and node2vec/256, respectively. Similarly, AUPRCs and accuracies were obtained for the four different measurement standards. Clearly, node2vec showed the better performance when its feature dimension is set to 128 (Table 1), and the used default values of other hyperparameters are recommended in [21]. In addition, we also evaluate the performance of node2vec/256 with feature dimension of 256. Compared to node2vec/128, the performance was slightly improved, but because of the computational complexity and over-fitting caused by higher feature dimension, node2vec/256 was not taken into consideration, here. Next, in order to reveal the effectiveness of our model, we would implement leave-one-out cross-validation (LOOCV) for HGCNMDA.

### 3.2. Performance of Model on Diseases

**Figure 5:**
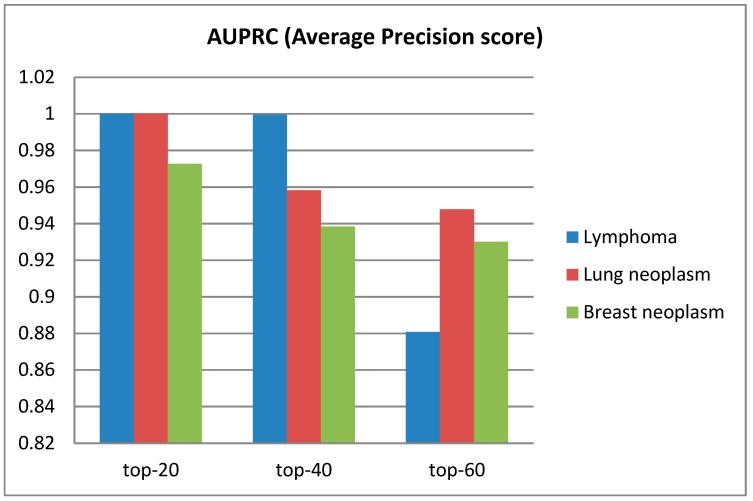
The average precision score (APs) for breast neoplasm, lymphoma, and lung neoplasm with top-20, top-40, and top-60, respectively.

**Figure 6:**
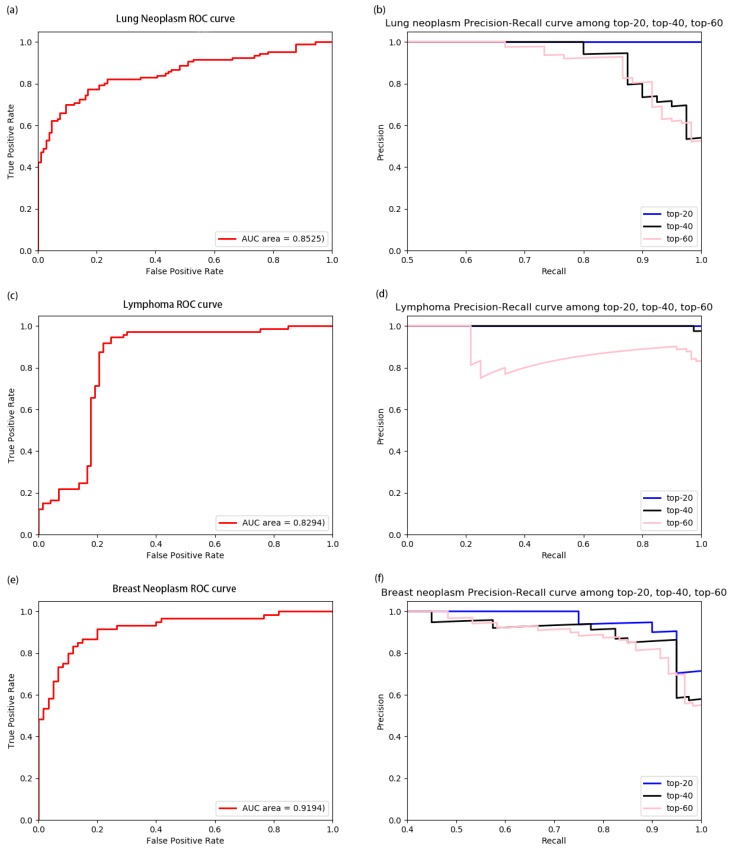
The ROC curves and precision–recall (PR) curves for lung neoplasm, lymphoma, and breast neoplasm with top-20, top-40, and top-60, respectively using leave-one-out cross-validation LOOCV. (**a**) and (**b**) showed us the ROC curve and PR curve for lung neoplasm; (**c**) and (**d**) was the ROC curve and PR curve of lymphoma; (**e**) and (**f**) showed the ROC curve and PR curve for breast neoplasm. Performances of predicting top-20, top-40, top-60 for the three diseases mentioned above were compared in terms of ROC curves and PR curves.

In order to evaluate the validity of HGCNMDA, we used this model to predict disease-related miRNAs for some specific diseases in terms of average precision score (APs) and AUROC score as a measure. Figure 5 shows the average precision score (APs) predicted by HGCNMDA for breast neoplasm, lymphoma, and lung neoplasm in terms of top-20, top-40, and top-60, respectively. We chose these three diseases because they have more than 60 known associated with miRNAs. From this histogram, the three diseases in top-20 prediction achieved the best performance, while slightly worse performances were achieved in top-60. The larger the APs value, the better the performance. Furthermore, the ROC curves based on leave-one-out cross-validation LOOCV and precision-recall curves for the three diseases mentioned above were plotted by Figure 6. For more details, please refer to Figures S2, S3 and S4. HGCNMDA achieved reliable performance with an average AUC of 0.8671 based on LOOCV for breast neoplasm, lymphoma, and lung neoplasm diseases. As k of top- k increases, recall is on the rise, but precision decreases. As showed in the PR curves, the AUROC of lung neoplasm was 0.8525, a better AUROC value of 0.9194 was obtained in predicting breast neoplasm. Obviously, our method achieved good average performance. However, PR curve of lymphoma in top-60 deviated greatly from top-40. As we know, precision = TP/TP + FP = 1/(1 + FP/TP), recall = TP/TP + FN = 1/(1 + FN/TP), where TP is the count of true positive samples, FP is the count of false positive samples and FN is the count of false negative samples. When the number of positive samples decreases, FN and TP decrease, while FP increases, so FN/TP decreases slightly and FP/TP increases sharply. Therefore, recall score increases slightly, while precision score decreases sharply. In the case of data imbalance, the PR curve is sensitive, and the PR curve will change strongly as the ratio of positive and negative samples changes. Among these three diseases, due to less association positive instances in top-60 than top-40 for lymphoma, Figure 6d shows the abrupt deviation in PR curve of the lymphoma case.

### 3.3. Comparison To Other Algorithms

**Figure 7:**
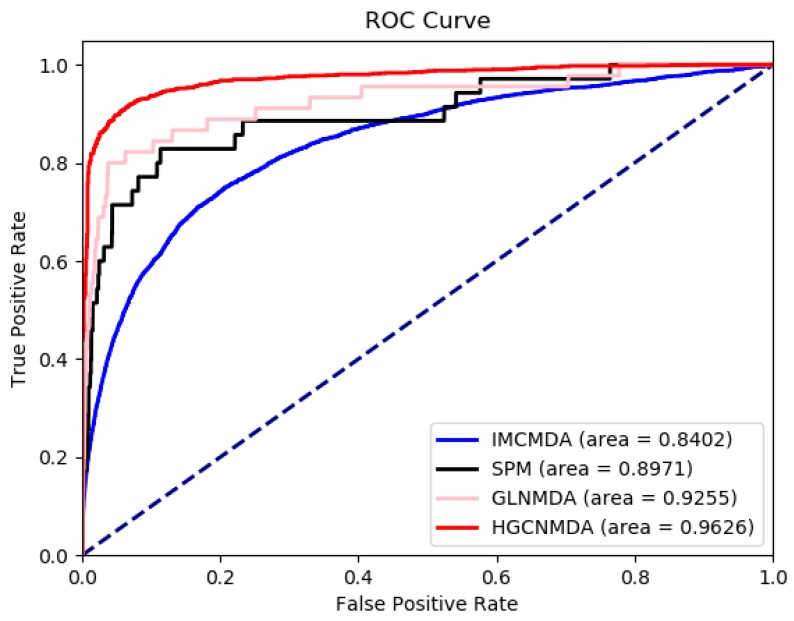
ROC curves between HGCNMDA and baseline methods: inductive matrix completion method (IMCMDA), structural perturbation method (SPM), and Global linear neighborhoods method (GLNMDA). HGCNMDA outperforms the previous methods with an AUC of 0.9626.

HGCNMDA was further compared to three newly developed algorithms: IMCMDA [9], GLNMDA [10], and SPM [12], all of which have obtained excellent performance for prediction of latent disease-related miRNAs. However, they are based on similarity method. The AUC scores were obtained with 5-fold cross-validation, respectively, which of four competing algorithms were shown in Figure 7. The horizontal axis is false positive rate (FPR), the vertical axis is true positive rate (TPR). FPR = FP/TN + FP, TPR = TP/TP + FN, where TP, FP, FN, and TN denote the number of true positive samples, false positive samples, false negative samples, and true negative samples, respectively. Different colors represent different algorithms. Red curve is the HGCNMDA algorithm we proposed. From the ROC curve, HGCNMDA achieved an AUC of 0.9626 which was the best performance in four competitive algorithms. The AUC of GLNMDA was 0.9255, which is slightly worse than that of HGCNMDA. The AUC of SPM was 0.8971. IMCMDA ranked the fourth with an AUC of 0.8402. Obviously, HGCNMDA consistently outperformed the methods using 5-fold cross-validation. On the other hand, we notice that IMCMDA and HGCNMDA have smooth ROC curves, whereas SPM and GLNMDA have serrated curves. The main reasons for this were differences in sample size, data imbalance, and different definitions of thresholds, but the overall performance was identical. All in all, HGCNMDA can be used as a reliable model for predicting the latent miRNA–disease interactions.

### 3.4. Prediction of New miRNA–Disease Associations

In order to further validate the predictive ability of HGCNMDA, we implemented two case studies of human diseases based on the correlation probability calculated by HGCNMDA. The first case study was implemented for osteosarcoma (OS). It is reported that osteosarcoma is a cancerous tumor in the skeleton. It is an aggressive malignant tumor, originating from mesenchymal primitive transforming cells, which manifests osteoblastic differentiation and produces malignant osteoid [49]. Here, we ranked the top-10 predicted miRNAs in the disease based on the unknown miRNA–disease interactions. In this experiment, the known miRNA–disease associations not included in HMDD v3.0 and miR2Disease were used to validate the performance of HGCNMDA. Finally, 8 of top-10 predicted miRNAs were verified to be related with the specific disease. We conducted the second case study for polycystic ovary syndrome (PCOS). This is a common hormone disorder in women of reproductive age. Women with PCOS may have very short or prolonged menstruation or high levels of male hormone. The ovaries may develop large amounts of fluid that do not release eggs regularly. We used HGCNMDA to forecast the latent relevance between PCOS and miRNAs. As a result, 7 of top-10 predicted latent miRNAs have been verified by relevant literatures. Three unconfirmed miRNAs were hsa-mir-34a, hsa-mir-126, and hsa-mir-210, respectively. In addition, the accuracy of top-5 prediction was 100%. Our predictions were consistent with the existing research results. The top-10 OS and PCOS-associated miRNAs prediction using our method were listed in Table 2.

## 4. Discussion and Conclusions

Integrating multiple types of data by using a deep-learning model is a challenging job, especially in prediction of disease-related miRNAs with a limited number of known associations. In this paper, we proposed a novel model to forecast miRNA–disease interactions in heterogeneous networks, namely miRNAs–disease networks, diseases networks, miRNAs networks and PPI networks. First of all, the HGCNMDA model extracted raw features based on PPI networks. Then, HGCNMDA model learned feature representations based on graph convolutional network by combining graph structure features and latent features. Lastly, edge features extraction component of HGCNMDA was built for predicting the edge type. Experiments demonstrated that HGCNMDA had reliable performance in top-r predictions (r equals 20, 40, or 60, respectively) for breast neoplasm, lymphoma, and lung neoplasm based on LOOCV. Compared to other competing algorithms, results demonstrated that HGCNMDA is much more powerful for predicting disease-associated miRNAs. Furthermore, two case studies on OS and PCOS were conducted and HGCNMDA achieved a good performance. Therefore, HGCNMDA is an effective model for predicting potential miRNA–disease interactions.

The success of HGCNMDA is mainly due to the following factors. First, we propose this completely novel method HGCNMDA to predict disease–miRNA interactions, taking the PPI network as a medium to avoid the complexity of constructing similarity-based methods. Therefore, our model is concise and easy to use. Second, using a deep-learning approach in heterogeneous environments, feature extraction and link prediction are simultaneously employed by one pipeline instead of a two-stage pipeline. Third, the known experimentally verified miRNA–disease interactions are used as the benchmark in terms of the cross-validation. Lastly, a new approach to sampling negative samples is taken to increase the model robustness during training.

Nevertheless, current HGCNMDA models still have some limitations. First, the model can be made better according to integrating more known human miRNA–disease interactions. Second, multi-view original feature can be properly integrated, for example, disease attribute characteristics and miRNA structure features can be constructed and learned to accurately obtain joint feature representations and thus enhance performance. We will leave this issue for future work.

## Figures and Tables

**Table 1:** The scores of accuracy, AUROC, AUPRC for one-hot, node2vec/64, node2vec/128, and node2vec/256.

Model Baselines	Accuracy	AUROC	AUPRC
HGCNMDA (One-hot)	0.8497	0.9036	0.8776
HGCNMDA (Node2vec/64)	0.8626	0.9358	0.9290
HGCNMDA (Node2vec/128)	0.9108	0.9626	0.9660
HGCNMDA (Node2vec/256)	0.9116	0.9651	0.9689

**Table 2:** Top-10 osteosarcoma and polycystic ovary syndrome-associated miRNAs.

Osteosarcoma		Polycystic Ovary Syndrome	
miRNA	Evidence	miRNA	Evidence
hsa-mir-26b	dbDEMCv2.0	hsa-mir-9	(Sørensen et al., 2014) [50]
hsa-mir-218	Unconfirmed	hsa-mir-21	(Sørensen et al., 2014) [50]
hsa-mir-873	Unconfirmed	hsa-mir-155	(Sørensen et al., 2014) [50]
hsa-mir-383	dbDEMCv2.0	hsa-mir-146a	(Sørensen et al., 2014) [50]
hsa-mir-16	dbDEMCv2.0	hsa-mir-223	(Chuang et al., 2015) [51]
hsa-mir-199a	dbDEMCv2.0	hsa-mir-34a	Unconfirmed
hsa-mir-671	dbDEMCv2.0	hsa-mir-145	(Cai et al., 2017) [52]
hsa-mir-367	dbDEMCv2.0	hsa-mir-126	Unconfirmed
hsa-mir-145	dbDEMCv2.0	hsa-mir-210	Unconfirmed
hsa-mir-17	dbDEMCv2.0	hsa-mir-32	(Roth et al., 2014) [53]

## Supplementary Materials

The following are available online at https://www.mdpi.com/2073-4409/8/9/977/s1, Figure S1: The distribution of 7040 positive samples of training neural network which are associated with 390 diseases and 567 miRNAs. The horizontal axis represents the type of disease and the vertical axis represents the number of miRNAs associated with the disease; Figure S2: Precision-recall curves and AUPRC for lung neoplasm with top-40 and top-60, respectively. (a) PR curve and APs for top-40 prediction; (b) PR curve and APs for top-60 prediction; Figure S3: Precision-recall curves and AUPRC for lymphoma with top-40 and top-60, respectively. (a) PR curve and APs for top-40 prediction; (b) PR curve and APs for top-60 prediction; Figure S4: Precision-recall curves and AUPRC for breast neoplasm with top-40 and top-60, respectively. (a) PR curve and APs for top-40 prediction; (b) PR curve and APs for top-60 prediction.
