# Supplementary material for: A Novel Computational Model for Predicting microRNA–Disease Associations Based on Heterogeneous Graph Convolutional Networks
Source: Cells. 2019 Aug 26;8(9):977. doi: 10.3390/cells8090977 (PMC6769654; doi:10.3390/cells8090977)
Supplement: Supplementary file 1 [file cells-08-00977-s001.zip › cells-567004-supplementary/supplementary/supplementary materials.docx]

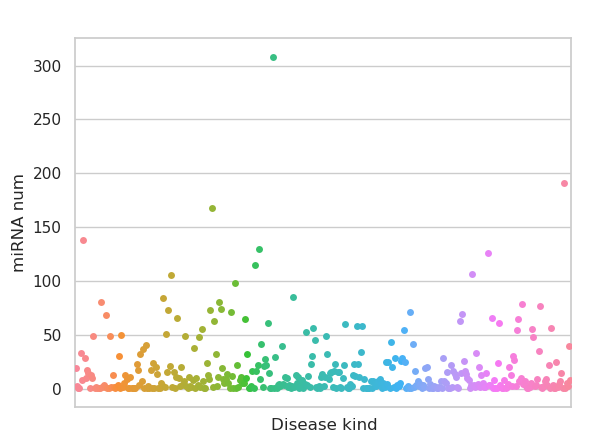


**Figure S1.** The distribution of 7040 positive samples of training neural network which are associated with 390 diseases and 567 miRNAs. the horizontal axis represents the type of disease and the vertical axis represents the number of miRNAs associated with the disease.

| 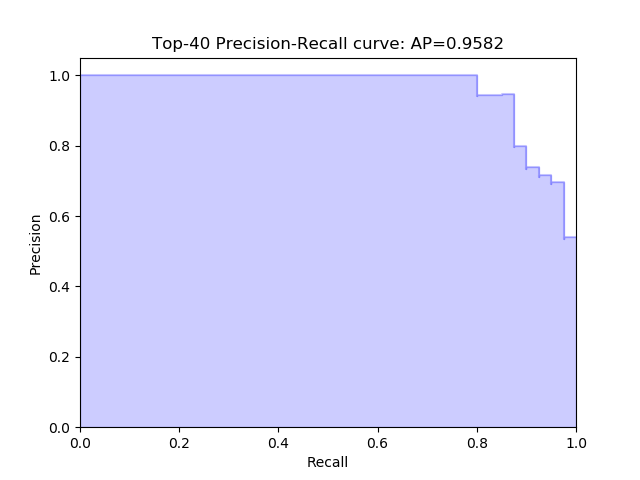  (**a**) | 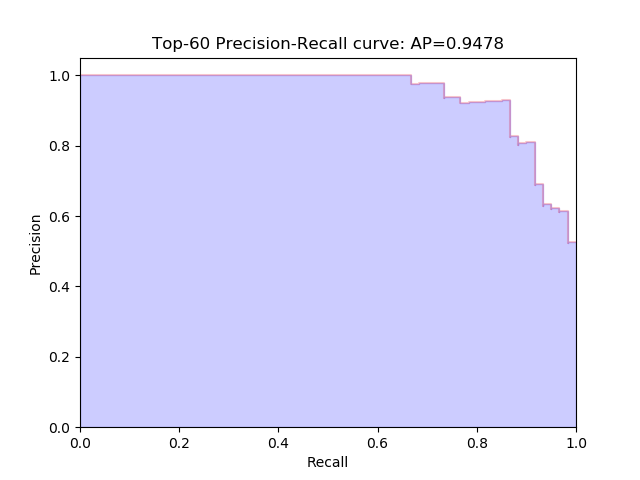  (**b**) |
| --- | --- |

**Figure S2.** Precision-Recall curves and AUPRC for Lung Neoplasm with top-40 and top-60, respectively. (**a**) PR curve and APs for top-40 prediction; (**b**) PR curve and APs for top-60 prediction.

| 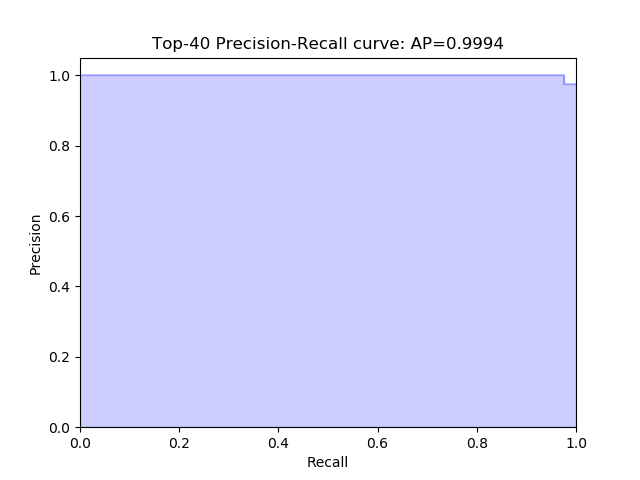  (**a**) | 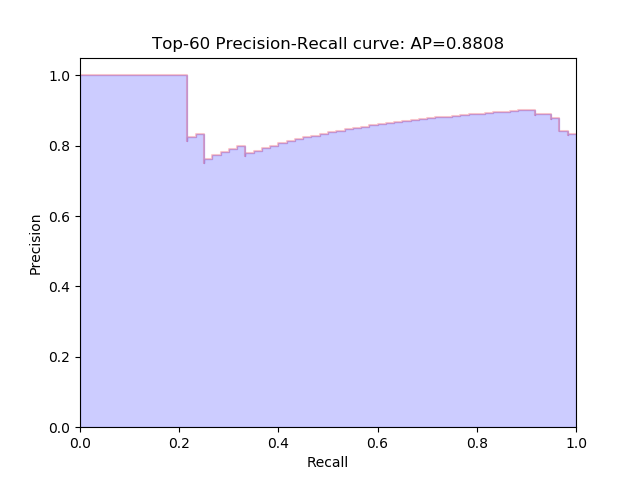  (**b**) |
| --- | --- |

**Figure S3.** Precision-Recall curves and AUPRC for Lymphoma with top-40 and top-60, respectively. (**a**) PR curve and APs for top-40 prediction; (**b**) PR curve and APs for top-60 prediction.

| 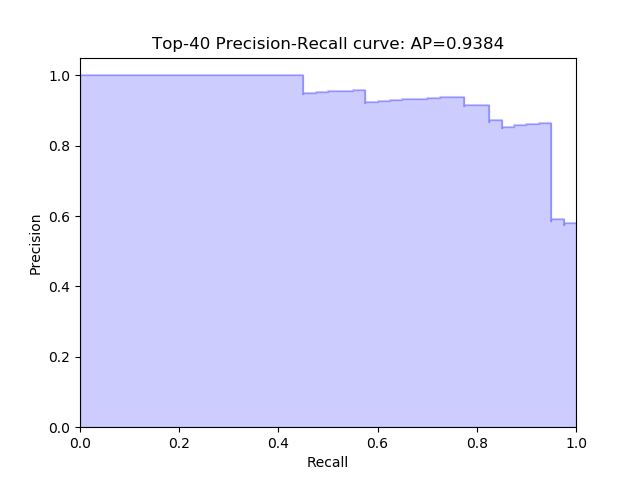  (**a**) | 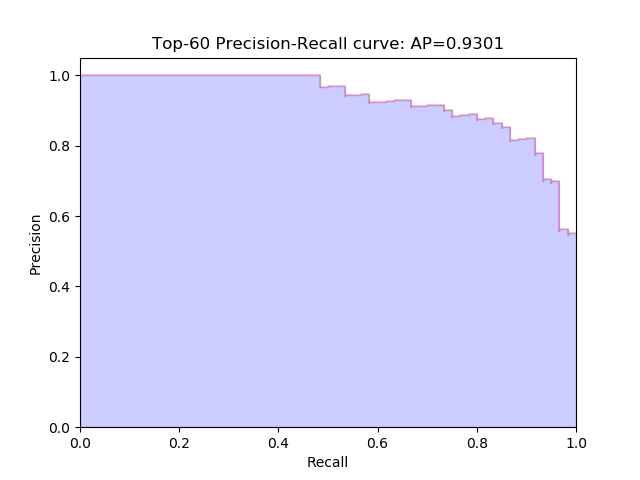  (**b**) |
| --- | --- |

**Figure S4.** Precision-Recall curves and AUPRC for Breast Neoplasm with top-40 and top-60, respectively. (**a**) PR curve and APs for top-40 prediction; (**b**) PR curve and APs for top-60 prediction.
