# Supplementary figures and images for: A Novel Computational Model for Predicting microRNA–Disease Associations Based on Heterogeneous Graph Convolutional Networks
Source: Cells. 2019 Aug 26;8(9):977. doi: 10.3390/cells8090977 (PMC6769654; doi:10.3390/cells8090977)

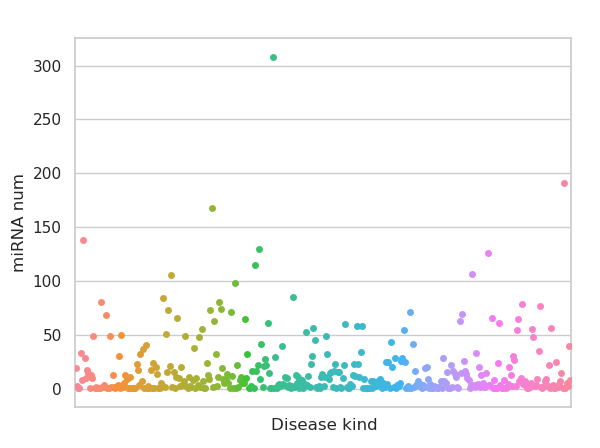

Supplement: Supplementary file 1 [file cells-08-00977-s001.zip › cells-567004-supplementary/supplementary/S1.png]

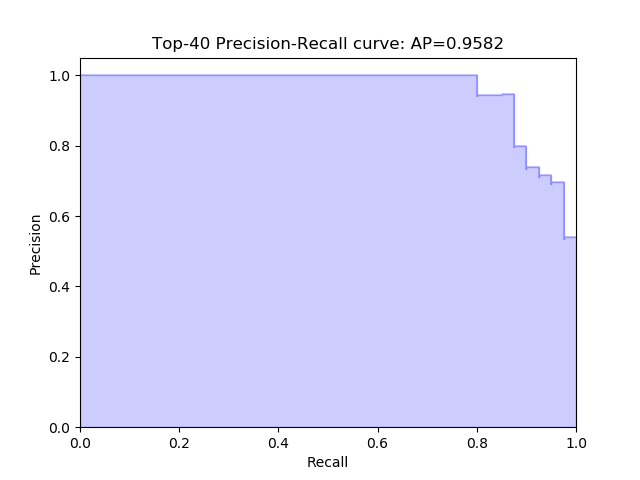

Supplement: Supplementary file 1 [file cells-08-00977-s001.zip › cells-567004-supplementary/supplementary/S2-a.png]

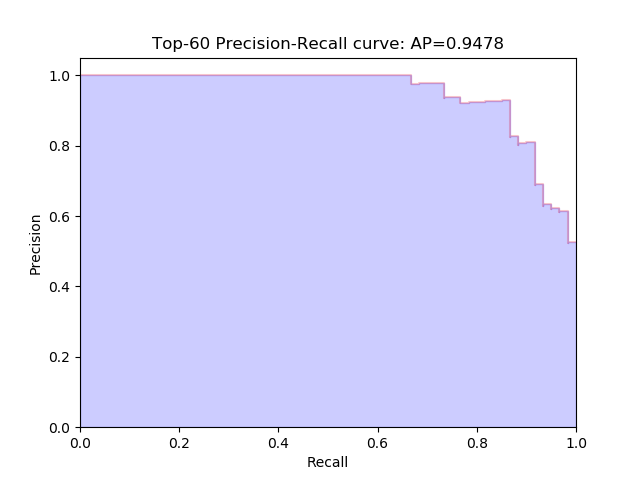

Supplement: Supplementary file 1 [file cells-08-00977-s001.zip › cells-567004-supplementary/supplementary/S2-b.png]

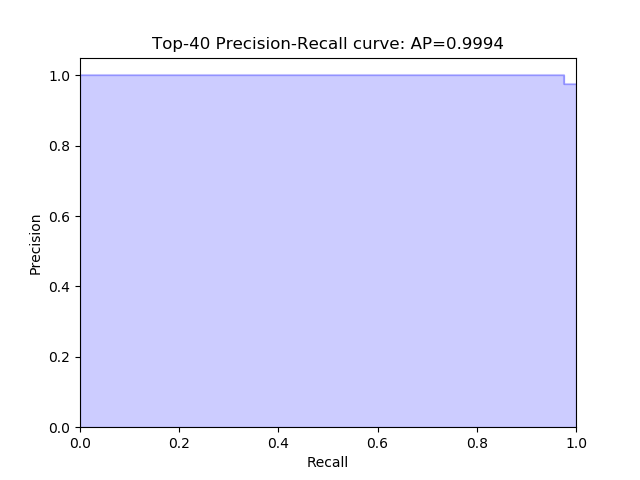

Supplement: Supplementary file 1 [file cells-08-00977-s001.zip › cells-567004-supplementary/supplementary/S3-a.png]

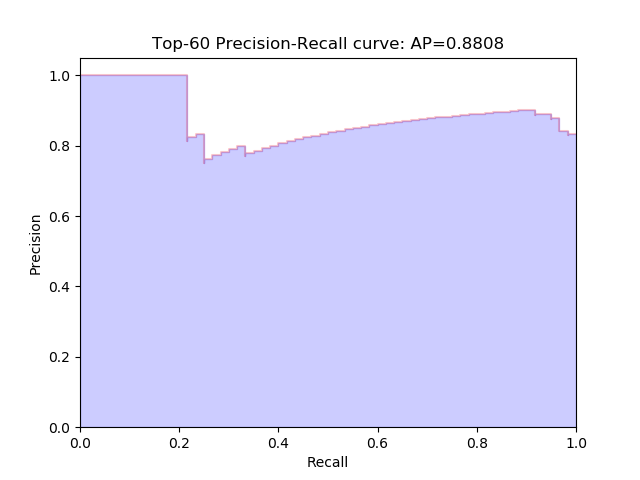

Supplement: Supplementary file 1 [file cells-08-00977-s001.zip › cells-567004-supplementary/supplementary/S3-b.png]

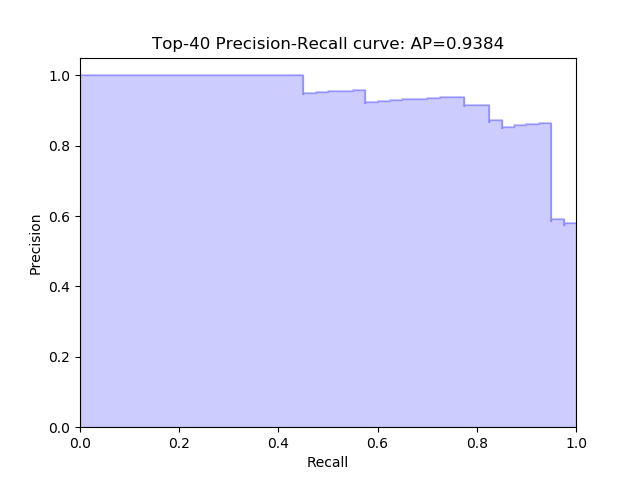

Supplement: Supplementary file 1 [file cells-08-00977-s001.zip › cells-567004-supplementary/supplementary/S4-a.png]

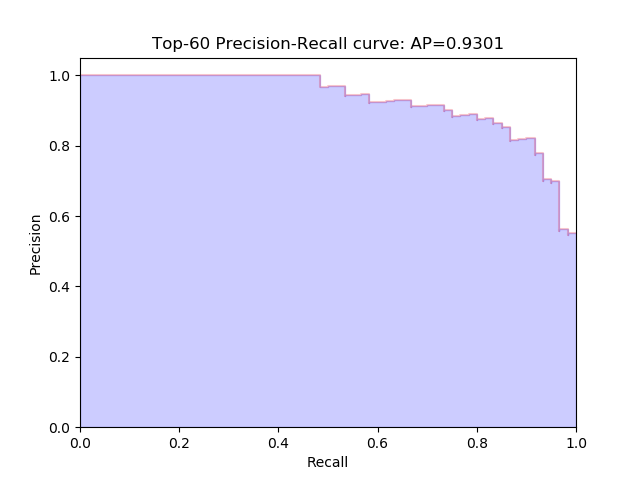

Supplement: Supplementary file 1 [file cells-08-00977-s001.zip › cells-567004-supplementary/supplementary/S4-b.png]
